# Etiology and Use of the “Hanging Drop” Technique: A Review

**DOI:** 10.1155/2014/146750

**Published:** 2014-04-15

**Authors:** Ludmil Todorov, Timothy VadeBoncouer

**Affiliations:** ^1^Department of Anesthesiology, Memorial Hospital of Rhode Island, Alpert Medical School, Brown University, 111 Brewster Street, Pawtucket, RI 02860, USA; ^2^Department of Anesthesiology, University of Illinois at Chicago College of Medicine, Jesse Brown VA Hospital, 820 S Damen Avenue, Chicago, IL 60612, USA

## Abstract

*Background*. The hanging drop (HD) technique presumably relies on the presence of subatmospheric epidural pressure. It is not clear whether this negative pressure is intrinsic or an artifact and how it is affected by body position. There are few data to indicate how often HD is currently being used. *Methods*. We identified studies that measured subatmospheric pressures and looked at the effect of the sitting position. We also looked at the technique used for cervical and thoracic epidural anesthesia in the last 10 years. *Results*. Intrinsic subatmospheric pressures were measured in the thoracic and cervical spine. Three trials studied the effect of body position, indicating a higher incidence of subatmospheric pressures when sitting. The results show lower epidural pressure (−10.7 mmHg) with the sitting position. 28.8% of trials of cervical and thoracic epidural anesthesia that documented the technique used, utilized the HD technique. When adjusting for possible bias, the rate of HD use can be as low as 11.7%. *Conclusions*. Intrinsic negative pressure might be present in the cervical and thoracic epidural space. This effect is more pronounced when sitting. This position might be preferable when using HD. Future studies are needed to compare it with the loss of resistance technique.

## 1. Introduction

**Figure 1:**
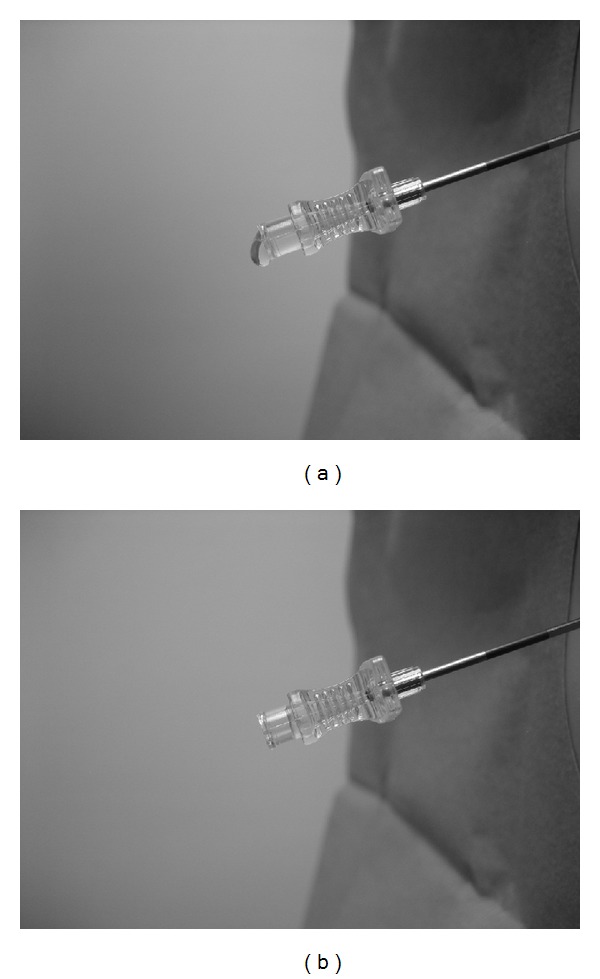
(a) A small volume of fluid is injected into the hub of the needle. (b) As the needle is advanced into the epidural space, the pressure at the tip decreases below atmospheric and the fluid is aspirated from the hub.

Epidural anesthesia is popular in the treatment of acute and chronic pain. Most commonly the “loss of resistance” (LOR) technique is used. An alternative method is the “hanging drop” (HD) technique. It relies upon the aspiration of a small volume of fluid from the hub of the needle as the pressure at the tip decreases below atmospheric level upon entry into the epidural space (Figure 1 and Video, Supplemental Digital Content 1 in Supplementary Material available online at http://dx.doi.org/10.1155/2014/146750, demonstrating the HD technique). Even though the technique has been used for 80 years there are still questions that remain unanswered. It is not clear whether the negative pressure required for entry into the epidural space is intrinsic or an artifact and how it is affected by body position. In addition, there are very few data to indicate how often this technique is currently used. The primary objective of this systematic review was to identify studies that measured subatmospheric pressures in the epidural space and to determine how these compared in the sitting versus the prone or lateral decubitus position. We also tried to determine how often the HD technique has been used in clinical trials in the last 10 years.

## 2. Methods

We performed a search of the databases MEDLINE, CINAHL, Turning Research into Practice (TRIP), and the Cochrane Library using the terms: Epidural Space/physiology [Mesh] or hanging drop and epidural or subatmospheric epidural pressure or negative epidural pressure. The search was limited to publications in English and humans. Since epidural anesthesia is usually performed in awake, spontaneously breathing patients, studies performed under general anesthesia and positive pressure ventilation were excluded. Case reports, reviews, editorials, practice guidelines, comments, and letters to the editor were also excluded. The reference sections of the articles that fulfilled the inclusion criteria were examined for any relevant sources not identified in the initial search. All publications that fulfilled the eligibility criteria were screened to identify trials demonstrating the presence of intrinsic subatmospheric epidural pressure and studying the effect of body position.

To identify how often the HD technique is used we searched MEDLINE using the terms: thoracic epidural or cervical epidural and anesthesia. The search was limited to clinical trials in human adults in the last 10 years and English language.

### 2.1. Data Extraction

The reviewers independently reviewed the full manuscripts of all included trials. The data extracted included the number of patients, epidural pressures, and body position. The timing of pressure measurements was also recorded. Only those pressures that were measured with the needle (or catheter) stationary in the epidural space were considered to be intrinsic. This was done to exclude any negative pressure artifactsrelated to the interaction of the advancing needle with the ligamentum flavum and the dura.When assessing the intrinsic epidural pressures, the measurements were identified as positive (above atmospheric), atmospheric, or negative (below atmospheric) without considering the pressure magnitude. When studying the effect of body position, the mean and standard deviation of the measured epidural pressures, as well as the incidence of subatmospheric pressure, were recorded. For the second search, each article was screened and the results were recorded as HD, LOR, both (LOR and HD), or unknown if the technique of epidural cannulation could not be identified from the text.

### 2.2. Risk of Bias Assessment

Risk of bias assessment was performed for those publications that demonstrated the presence of intrinsic subatmospheric epidural pressure. For randomized controlled studies, we used the 12 criteria established by the Cochrane Back Review Group (CBRG) [1]. These are presented in Table 1. Studies are rated as having a “high risk” of bias if less than 50% of CBRG criteria have been met. For all other studies, we used a modification of the Methodological Index for Non-Randomized Studies (MINORS) [2]. This tool contains 8 items with 4 additional items for comparative studies. Items can be scored as “adequate,” “inadequate,” or “unclear” if there is insufficient information (Table 2). To reduce the chance of publication bias, multiple clinical trial registries were screened for evidence of missing information (Table 3).

### 2.3. Statistical Analysis

Meta-analysis was used to calculate the pooled effects of the sitting versus the horizontal position (prone or lateral decubitus). Analyses were performed using RevMan 5.2 software (Cochrane Collaboration, Oxford, UK). When looking at epidural pressures, the results were expressed as mean differences with 95% confidence intervals using the random effects model. Odds ratios with 95% confidence intervals were calculated using the Mantel-Haenszel random effects model when assessing the incidence of subatmospheric pressures. Statistical heterogeneity of the included studies was determined using the *I* ^2^ statistic which describes the degree of total variation between studies that cannot be explained by chance alone. Values <25% indicate low levels, whereas values >50% are consistent with substantive heterogeneity.

## 3. Results

**Figure 2:**
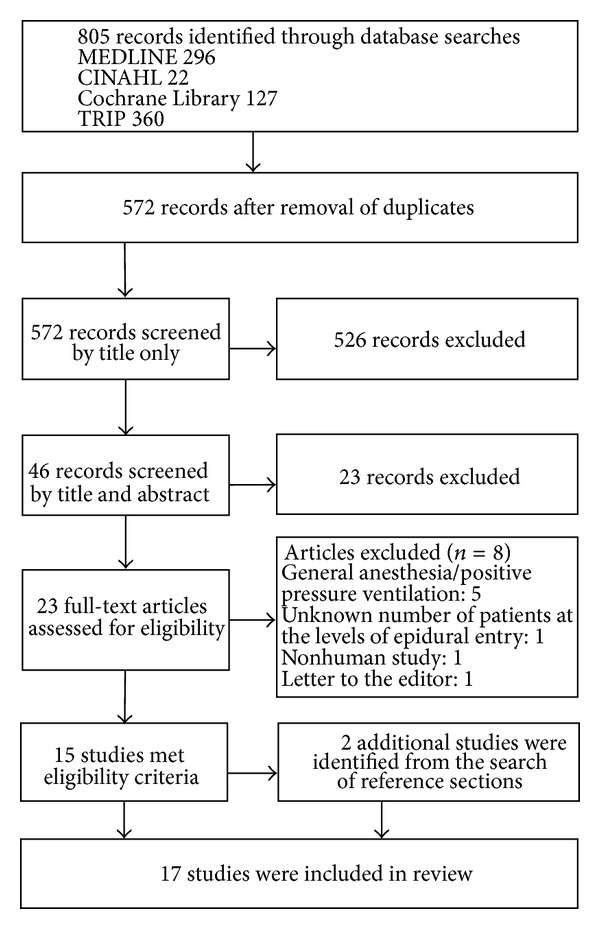
Flow diagram of the literature search results on epidural pressures.

A total of 17 article publications were identified that matched our criteria [3–19]. The initial search identified 296 records in MEDLINE, 22 in CINAHL, 127 in the Cochrane library, and 360 in TRIP (Figure 2). The titles were examined and those that were not considered relevant to our search were discarded. After reviewing the abstracts and full-text articles, a total of 15 studies were identified that matched the inclusion criteria with a total of 802 patients. A search of the references identified 2 additional articles with a total of 28 patients [7, 17]. 13 studies reported on pressures in the lumbar spine, 5 in the thoracic spine, and 3 in the cervical spine. A brief summary of the main findings is listed in Table 4. In the lumbar spine, subatmospheric pressures were only measured at the time of needle entry into the epidural space. In those cases where the needle was stationary or the pressures were measured using an epidural catheter, the pressures were always positive. This was true independent of patient position. In the thoracic spine, subatmospheric pressures were measured in all 5 studies. Only three studies measured intrinsic epidural pressures after needle stabilization [3, 6, 18]. In all three, epidural pressures were measured with the patient in the lateral decubitus position and even though there were instances of negative epidural pressures measured at least 90 seconds after needle entry, the mean epidural pressures were positive (1 mmHg at T3–5, 5.1 mmHg at T5-6, and 3.7 mmHg at T7-8) [3, 6, 18]. Consistent intrinsic negative pressure measurements were only found in the one study in which the sitting position was also chosen [3]. In all 3 studies that focused on the cervical spine, negative pressures were measured [4, 5, 17]. The recordings were made after needle stabilization only in one study [4]. Moon et al. consistently measured positive pressures with the patients in the prone position, whereas 10 out of 15 measurements were subatmospheric when sitting [4].

**Figure 3:**
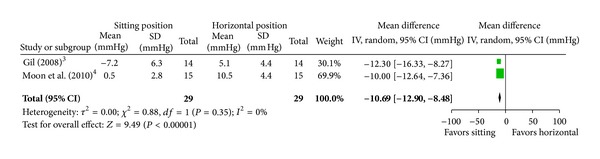
Pooled data evaluating the difference in epidural pressure (mm Hg) between the sitting and horizontal position. Expressed as mean difference with 95% confidence intervals.

**Figure 4:**
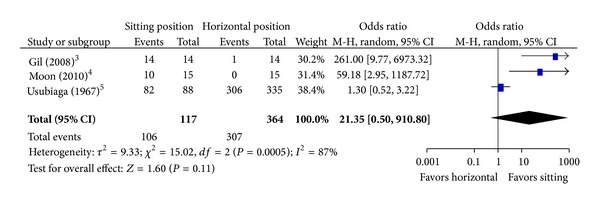
Effect of the sitting position on the incidence of subatmospheric pressure. Expressed as pooled odds ratio with 95% confidence intervals. The 95% confidence interval crosses “1,” suggesting that there is no difference between the sitting and horizontal position.

When looking at those publications where negative pressures were measured, 3 studies compared the sitting position to the lateral decubitus or prone position. All three (one cervical, one thoracic, and one with cervical, thoracic, and lumbar measurements) studies showed a greater incidence of measured subatmospheric pressures when sitting [3–5]. Table 1 summarizes the risk of bias assessment of these trials. Our search of trial registries did not reveal any unpublished trials. There was no evidence of missing data upon close examination of the articles and in one case upon examination of the study protocol [4]. The data from the largest study by Usubiaga et al. [5] were only presented as range of measured epidural pressures and incidence of subatmospheric pressure which made it impossible to calculate the mean and standard deviation. The results of the two remaining studies were combined in a forest plot of epidural pressures (Figure 3). The combined effect of the meta-analysis was −10.7 mm Hg mean difference (95% confidence interval −12.9 to −8.5) suggesting a lower epidural pressure in the sitting position that was statistically significant (*P* < 0.0001). When looking at the incidence of subatmospheric pressure, all 3 studies were included in the meta-analysis (Figure 4). The pooled effect showed no difference between the two positions. There was high heterogeneity (*I* ^2^ = 87). A funnel plot was not done due to the small number of studies.

The second MEDLINE search using the terms: “thoracic epidural or cervical epidural and anesthesia” revealed 209 articles. After discarding articles that were not relevant for reasons such as levels other than thoracic or cervical, no epidural technique used, or retracted publications, either the abstract or full text of the remaining articles was used to identify the technique of epidural cannulation. 78 studies did not indicate what technique was used, in 47 studies only the LOR technique was used, in 14 studies only HD was used, and 5 studies used both techniques. This gives a rate of 28.8% (19/66) for the HD technique in those published controlled studies in the last 10 years where the technique could be identified from the article. Those studies that utilized the HD technique are listed in Table 5 [20–38]. To avoid any bias due to multiple publications from the same source, the articles were screened, and in all cases where several articles came from the same institution or the same authors, they were counted as one. The revised numbers were LOR: 43, HD: 11, and both: 5, giving an overall rate of 27.1% (16/59) for HD in the last 10 years. The total number of patients receiving epidural anesthesia in these trials was 3319 (LOR-1855, HD-485, both-979). To avoid any bias due to the omission of the 78 studies that did not mention the technique of epidural cannulation, it was assumed that they all used LOR, thus allowing us to calculate the lowest possible rate of HD use, namely, 11.7% (16/137). The majority of studies focused on the thoracic spine, while only two were performed in the cervical spine (1 LOR and 1 with both LOR and HD). All studies that used the HD technique came from continental Europe and Asia.

## 4. Discussion

Our review of the literature suggests that intrinsic negative pressures might be present in the cervical and thoracic epidural space. Subatmospheric pressures are more pronounced when the sitting position is chosen. We also found that the HD technique was used in 28.8% of those controlled trials of cervical and thoracic epidural anesthesia in the last 10 years that documented the method of cannulation.

### 4.1. Etiology of the Negative Epidural Pressure

In 1933, Guttierez described the sign of the “hanging drop,” whereby a drop of saline hanging in the hub of a needle was “aspirated” when the needle entered the epidural space [39]. This phenomenon presumably occurs due to the presence of subatmospheric pressure at the needle tip. In 1926, Ernst Janzen was the first to describe the presence of subatmospheric (negative) pressure in the epidural space [40]. There has been controversy whether there is intrinsic negative epidural pressure or if it is an artifact produced by the needle entering the epidural space [40–43]. Our literature search suggests that there is no evidence of intrinsic subatmospheric pressure in the lumbar epidural space that can be measured after needle stabilization. The negative pressures recorded at the time of lumbar epidural entry have been described as artifacts caused by the initial bulging of the ligamentum flavum, followed by its rapid return to the resting position once the needle has perforated the ligament or due to tenting of the dura by the advancing needle [19, 40, 44]. Our literature search suggests that intrinsic subatmospheric epidural pressure might be present in the human thoracic and cervical spine. This was the case in all 4 studies (3 thoracic and 1 cervical) that measured epidural pressures after needle stabilization. In the studies identified by our systematic search, we only found evidence of consistent intrinsic negative pressure in the sitting position in the thoracic spine, based on the observations in one report [3]. A possible explanation for this phenomenon is that blood in the epidural venous plexus may be distributed to the lower part of the body due to gravity and the volume of the epidural plexus may decrease, producing a lower epidural pressure. CSF in the dural sac may play a similar role [3]. Intrinsic subatmospheric pressure is not present consistently in the sitting position in the cervical spine and this might be caused by neck flexion, which is necessary to widen the interlaminar space during needle placement. This can prevent venous run-off and cause venous engorgement resulting in a decrease in the epidural volume and increase in pressure [4, 17]. Neck flexion can also cause compression of the jugular veins resulting in increased cerebrospinal fluid pressure, which in turn raises the epidural pressure [45].

Why would the existence of intrinsic subatmospheric pressure be important? It is not a requirement for the successful use of the hanging drop technique due to the occurrence of negative pressure artifacts [46–49]. The existence of intrinsic subatmospheric pressure may be important for the identification of the epidural space in those areas that have ligamentum flavum gaps or where dural tenting is limited by very small amounts of CSF and the presence of the spinal cord, which could potentially make negative pressure artifacts less reliable.

### 4.2. Comparison to Animal Studies

Negative epidural pressures have also been measured in dogs, horses, and cows [50–54]. Some of the animal data were recorded from chronically implanted (7–14 days) epidural catheters [50]. It is believed that such a time period would allow healing and resolution of any changes related to the needle trauma and produce measurements representing the intrinsic epidural pressure [50]. In all these studies, the pressures were either measured in standing animals (cows, horses) [51–54] or animals placed on their sternum (dogs) [50]. Such positioning produces negative intraabdominal pressure, probably related to the gravity of the organs in the abdominal cavity. The negative pressures are then transmitted to the epidural space through the intervertebral foramina [52]. Changing the body position in cattle from standing to lateral recumbent changes the mean lumbar epidural pressures from negative to positive [54]. This might explain why there is a positive “hanging drop” sign in 88% of dogs positioned on their sternum as opposed to 0% in those that are in the lateral recumbent position [55]. In human studies, only Shah et al. looked at the effect of the prone position in 5 pregnant patients on their hands and knees and showed that the pressures were lower, yet still positive in comparison to the supine and lateral positions (+2.2 cm H_2_O prone, +14.8 cm H_2_O lateral, and +22.6 cm H_2_O supine) [13]. The above-mentioned animal studies did not look at thoracic or cervical epidural pressures. Only Nystrom et al. looked at thoracic pressures in pigs [56]. The epidural pressures were consistently positive; however, they were measured in the lateral recumbent position and the animals were mechanically ventilated.

### 4.3. Effect of the Sitting Position

The three studies that were analyzed demonstrated a higher incidence of negative epidural pressures with the sitting position. When the data was pooled, there was a statistically significant decrease in epidural pressure when sitting. This evidence is weak as it is only based on 2 randomized controlled trials with a total of 29 patients. Looking at the incidence of subatmospheric pressures allowed us to include a larger trial with 405 patients in the meta-analysis. Although the pooled results suggest that the incidence of subatmospheric pressures might not be increased by the sitting position, it is possible that methodological flaws in the study by Usubiaga et al. [5], including lack of randomization and inconsistencies in the methods of epidural pressure measurement, could have contributed to these findings. Further studies are needed to examine the effect of body position on epidural pressure and how this might affect the reliability of epidural space cannulation with the HD technique.

### 4.4. Use of the HD Technique

We found that the HD technique was used in 28.8% of those controlled trials of cervical and thoracic epidural anesthesia in the last 10 years that documented the method of cannulation. When accounting for multiple publications by the same institution and assuming that the studies that did not document the technique all used LOR, the rate decreases to 11.7%. This number is speculative but it represents the lowest possible rate for HD use from the included trials. However, even after such corrections, the results are still somewhat surprising when compared to the limited available information on the use of the HD technique, coming mainly from surveys of anesthesiologists. Wantman et al. carried out a survey of 1285 obstetric anesthesiologists in the United Kingdom regarding their preferred method of identifying the epidural space. When performing thoracic epidurals, 98% of the respondents chose the LOR method. 2% preferred alternative methods, including HD [57]. This study did not mention how often obstetric anesthesiologists perform thoracic epidurals and how their rate of HD use compares to the overall rate for anesthesiologists in the UK. In a survey of 617 Spanish anesthesiologists by Figueredo et al., 0.8% of respondents identified HD as the technique they most commonly use [58]. The overall rate of HD use was not mentioned. However, since this study identified only the most commonly used technique for epidural space cannulation, it did not account for the fact that some anesthesiologists, including the authors of this article, use both the LOR and HD techniques.

The articles that we identified in our search came from Europe and Asia. The greatest use of the hanging drop technique in the US might be in the field of chronic pain management. A national survey from 2002 demonstrated that this technique was used in 62% of academic centers, and 30% of private practices for cervical epidural steroid injections [59]. To ensure accuracy of placement and decrease patient discomfort, the routine use of fluoroscopy is recommended [60]. Most commonly, the prone position is chosen, so that both anteroposterior and lateral fluoroscopic images of the needle can be obtained [59–64]. In this position, intrinsic cervical epidural pressures are usually positive [4]. This could mean that a successful HD technique will have to rely on negative pressure artifacts, which might not occur due to ligamentum flavum gaps and the possibility of decreased dural tenting related to the presence of the spinal cord. Abram and Hogan suggested avoiding the HD technique with cervical epidural steroid injections based on 2 malpractice cases in which this method failed to reliably identify the epidural space [65].

Despite the long coexistence of the HD and LOR techniques, just two studies have directly compared them and only in the lumbar spine [46, 47]. Even though both techniques were equally successful in identifying the epidural space and there were no dural punctures, Hoffmann et al. showed that the tip of the epidural needle was 2.8 mm closer to the subarachnoid space with HD than with the LOR [46]. Both studies were performed in nonobstetric patients. Many lumbar epidurals are performed in parturients who demonstrate increasing positive epidural pressures as labor progresses [7]. Janzen observed that increasing the abdominal pressure decreased the amplitude of the subatmospheric pressure during needle placement [40]. When performing lumbar and low (below the T8 level) thoracic epidural puncture, Bonica et al. observed a positive hanging drop sign in only 80% of cases [48]. In a study of 1002 single-shot lumbar epidural blocks by Sheehan et al., a positive sign occurred in 91% of cases [49]. It is therefore unlikely that using the HD technique in the lumbar spine will have any advantage over the LOR technique, and its use may be contraindicated because of the higher potential for failure. Future studies are needed to compare the safety and effectiveness of the LOR and HD techniques.

## 5. Limitations

We only pooled the data from the randomized trials. Pooling of both randomized and nonrandomized studies with the aim of performing meta-analysis of epidural pressures would have been difficult due to the fact that the studies performed measurements at different levels in the spine which can account for significant variations. Due to the limited number of publications that identified intrinsic subatmospheric epidural pressures, we looked at data from animal studies. The meta-analysis of the effect of the sitting position includes only three studies with the largest one demonstrating significant methodological flaws and a high risk of bias. The limited number of studies did not allow us to use methods such as funnel plots or formal testing to look for publication bias. Instead, we examined multiple registries of clinical trials for missing data. Future studies are needed to confirm our findings.

With respect to the use of the hanging drop technique, we are aware of the limitations of our findings due to the fact that 78 out of 144 articles that were screened did not mention the technique used. There might also be bias towards academic institutions, and thus not reflecting the overall use of the HD technique. We only chose the cervical and thoracic spine because we found no documented advantage of the HD technique over the LOR in the lumbar spine. In addition, we found very little evidence to suggest that the HD technique is routinely used in the lumbar spine. In the study by Hoffman et al., the cannulation was performed for the placement of intrathecal catheters for neurosurgical procedures [46]. The study by Gülen et al. used HD together with LOR in the control groups for the trial of the Episure spring-loaded syringe [47].

In conclusion, our review suggests that intrinsic negative pressure might be present in the cervical and thoracic epidural space. This effect is more pronounced when sitting, which is why this position might be preferable when using the HD technique. We found no information suggesting the presence of intrinsic negative pressure in the lumbar spine. There is also no evidence of any advantage of the HD technique over the LOR technique in the lumbar spine. If performing cervical epidural steroid injection with fluoroscopy in the prone position, the LOR technique should be used. Only studies directly comparing the LOR and HD techniques can determine which technique is better suited for the identification of the epidural space.

## Supplementary Material

Demonstration of the hanging drop technique. As the needle enters the epidural space the pressure at the tip decreases below atmospheric level. This results in aspiration of the fluid from the hub.

## Figures and Tables

**Table 1:** Risk of bias assessment using the CBRG criteria.

	Gil et al. (2008) [3]	Moon et al. (2010) [4]	Usubiaga et al. (1967) [5]
Was the method of randomization adequate?	Yes	Yes	No
Was the treatment allocation concealed?	No	No	No
Was the patient blinded to the intervention?	No	No	No
Was the care provider blinded to the intervention?	No	No	No
Was the outcome assessor blinded to the intervention?	No	Yes	No
Was the drop-out rate described and acceptable?	Yes	Yes	Yes
Were all randomized participants analyzed in the group to which they were allocated?	Yes	Yes	Yes
Are reports of the study free of suggestion of selective outcome reporting?	Yes	Yes	Yes
Were the groups similar at baseline regarding the most important prognostic indicators?	No	Unsure	No
Were cointerventions avoided or similar?	Yes	Yes	Unsure
Was the compliance acceptable in all groups?	Yes	Yes	Yes
Was the timing of the outcome assessment similar in all groups?	Yes	Yes	Yes

**Table 2:** Risk of bias assessment using MINORS.

	Visser et al. (2006) [18]	Okutomi et al. (1993) [6]
A clearly stated aim	A	A
Inclusion of consecutive patients	A	U
Prospective collection of data	A	A
Endpoints appropriate to the aim of the study	A	A
Unbiased assessment of the study endpoint	I	I
Follow-up period appropriate to the aim of the study	∗	∗
Loss to follow-up less than 5%	∗	∗
Prospective calculation of the study size	A	I
*Additional criteria for comparative studies *		
Adequate control group	A	
Contemporary groups	A	
Baseline equivalence of groups	A	
Adequate statistical analyses	A	

*No follow-up indicated. A: adequate; I: inadequate; U: unclear.

**Table 3:** Clinical trial registries that were screened for evidence of missing information.

U.S. National Institutes of Health	http://www.clinicaltrials.gov/
International Clinical Trials Registry Platform (ICTRP) of the World Health Organization	http://www.who.int/ictrp/en/
Australian New Zealand Clinical Trials Registry (ANZCTR)	http://www.anzctr.org.au/
Clinical Trials Registry-India	http://ctri.nic.in/
European Union Clinical Trials Register (EU-CTR)	https://www.clinicaltrialsregister.eu/
German Clinical Trials Register (DRKS)	http://www.drks.de/
Netherlands Trial Register	http://www.trialregister.nl/
International Standard Randomized Controlled Trial Number Register	http://www.isrctn.org/

**Table 4:** Summary of studies on epidural pressure.

Author (year)	Level	*n*	Epidural pressures	Comments
Galbert and Marx (1974) [7]	Lumbar	12	All positive	Pressures transduced from epidural catheter

Gil et al. (2008) [3]	Thoracic	28	Consistent negative epidural pressures in the sitting position only at T5-6	Pressures measured 120 s after entry into the epidural space

Johnston et al. (1989) [8]	Lumbar	14	All positive	Pressures transduced from epidural catheter

Messih (1981) [9]	Lumbar	21	All positive upon needle entry and after catheter insertion	Pressures measured in parturients

Moon et al. (2010) [4]	Cervical	30	All positive in the prone position, 10/15 negative in the sitting position	Pressures measured 120 s after entry into the epidural space

Okutomi et al. (1993) [6]	Thoracic	13	Initial negative pressure right after puncture, positive in 12/13 patients after 90 s.	Lateral decubitus position at T7-8

Rocco et al. (1997) [10]	Lumbar	25	All positive after needle was stabilized in the epidural space	negative in 4/4 patients where pressure was measured upon entry

Rodiera et al. (1995) [11]	Lumbar	20	All positive	Pressures measured >5 s. after entry into the epidural space

Shah (1981) [12]	Lumbar	43	All positive	Pressures transduced from epidural catheter

Shah (1984) [13]	Lumbar	40	All positive	Pressures transduced from epidural catheter

Takahashi et al. (1995) [14]	Lumbar	10	All positive	Measurements performed with a catheter transducer

Takahashi et al. (1995) [15]	Lumbar	19	All positive	Measurements performed with a catheter transducer

Thomas et al. (1992) [16]	Lumbar	39	All positive	Pressures measured 180 s after entry into the epidural space

Usubiaga et al. (1967) [17]	Lumbar	16	Consistent negative pressures measured at the time of entry into the epidural space, all positive pressure measurements after needle stabilization	Lateral decubitus position
	Thoracic			
	Cervical			

Usubiaga et al. (1967) [5]	Cervical	405	Consistent negative thoracic/cervical pressures in the sitting position	Pressures measured at the time of entry into the epidural space
	Thoracic		Negative lumbar pressures in 42/48 patients in the sitting position	
	Lumbar		Negative lumbar pressures in 202/228 patients in the lateral decubitus position	

Visser et al. (2006) [18]	Thoracic	40	Negative pressures in 8/17 patients at T3–T5 and 2/20 at T7–10 (lateral decubitus)	Pressures measured 120 s after entry into the epidural space

Zarzur (1984) [19]	Lumbar	30	Negative pressures in 24/30 patients upon entry into the epidural space	

Results are listed as either below (negative), or above (positive) atmospheric pressure.

**Table 5:** Summary of studies where the hanging drop technique was used.

Author	Year	Level	*n*	Patients receiving epidural anesthesia	Country of origin	Technique
Bauer et al. [20]	2007	Thoracic	68	34	France	HD
Berendes et al. [21]	2003	Thoracic	73	36	Germany	HD
Gupta et al. [22]	2006	Thoracic	60	30	Sweden	HD/LOR
Han et al. [23]	2003	Cervical	816	816	Korea	HD/LOR
Hansdottir et al. [24]	2006	Thoracic	113	58	Sweden	HD/LOR
Heijmans et al. [25]	2007	Thoracic	60	15	Netherlands	HD
Kessler et al. [26]	2005	Thoracic	90	60	Germany	HD
Kunstyr et al. [27]	2008	Thoracic	32	16	Czech Republic	HD
Kurtoğlu et al. [28]	2009	Thoracic	76	34	Turkey	HD/LOR
Lagunilla et al. [29]	2006	Thoracic	52	52	Spain	HD
Lundstrøm et al. [30]	2005	Thoracic	50	25	Denmark	HD
Mehta et al. [31]	2008	Thoracic	36	18	India	HD
Mehta et al. [32]	2010	Thoracic	62	31	India	HD
Nishi et al. [33]	2006	Thoracic	41	41	Japan	HD/LOR
Nygård et al. [34]	2004	Thoracic	163	79	Denmark	HD
Porizka et al. [35]	2011	Thoracic	47	32	Czech Republic	HD
Schmidt et al. [36]	2005	Thoracic	37	37	Germany	HD
Sharma et al. [37]	2010	Thoracic	60	30	India	HD
Visser et al. [38]	2006	Thoracic	20	20	Netherlands	HD

HD: hanging drop; LOR: loss of resistance.
